# Protocol: high-efficiency *in*-*planta Agrobacterium*-mediated transgenic hairy root induction of *Camellia sinensis* var. *sinensis*

**DOI:** 10.1186/s13007-018-0285-8

**Published:** 2018-02-23

**Authors:** Karthikeyan Alagarsamy, Lubobi Ferdinand Shamala, Shu Wei

**Affiliations:** State Key Laboratory of Tea Plant Biology and Utilization, Anhui Agricultural University, Hefei, 230036 Anhui China

**Keywords:** Hairy root, Tea, *Agrobacterium rhizogenes*, Polyphenols, Tissue browning, *In*-*planta*

## Abstract

**Background:**

*Camellia sinensis* var. *sinensis* is widely grown for tea beverages that possess significant health promoting effects. Studies on tea plant genetics and breeding are hindered due to its recalcitrance to *Agrobacterium*-mediated genetic transformation. Among the possible reasons, oxidation of phenolics released from explant tissues and bactericidal effects of tea polyphenols during the process of transformation play a role in the plant recalcitrance. The aim of the present study was to alleviate the harmful effects of phenolic compounds using *in*-*planta* transformation.

**Results:**

Two-month old seedlings of tea cultivar “Nong Kangzao” were infected at the hypocotyl with wild type *Agrobacterium rhizogenes* and maintained in an environment of high humidity. 88.3% of infected plants developed hairy roots at the wounded site after 2 months of infection. Our data indicated that transgenic hairy root induction of tea can be achieved using *A. rhizogenes* following the optimized protocol.

**Conclusion:**

With this method, composite tea plants containing wild-type shoots with transgenic roots can be generated for “in root” gene functional characterization and root-shoot interaction studies. Moreover, this method can be applied to improve the root system of composite tea plants for a better resistance to abiotic and biotic stresses.

## Background

Tea is consumed as a popular beverage globally. The People’s Republic of China ranks first (1,467,467 tonnes) in the world followed by India (991,180 tonnes), Kenya, Sri Lanka, and Turkey in tea production [1]. World tea production reached 4.52 million tonnes with an average consumption of 644.1 tonnes/day in the United Kingdom [2]. Tea is generally divided into pu-erh, oolong, green and black tea depending on the manufacturing process. In Asia pu-erh tea is almost exclusively consumed as compared to black tea which economically dominates the market and is popular in the West. In China and Japan green tea is common over the other types whereas, Oolong tea is preferred in some other countries [3].

Tea roots are rich in l-theanine, a unique non-protein amino acid synthesized in the roots, but accumulates in the leaves of tea plants. Recently l-theanine has been researched due to its beneficial effects on reducing anxiety, suppressing high blood pressure, improving learning ability, as well as promoting relaxation [4–8]. Tea leaves are rich in polyphenols, which exhibit anticancer, anti-allergic, antiviral, anti-inflammatory, antibacterial and immunostimulant effects [9]. The natural polyphenols may have beneficial antioxidant effects in humans due to their ability to deactivate free radicals within the body [10]. In particular, the various therapeutic properties of tea polyphenols have been explored recently for the development of novel antimicrobials to treat microbial infections [11, 12]. The most abundant flavanol group of polyphenols known as catechins present in green tea leaves are at 16–30% (DW), among which (−)-epigallocatechin gallate (EGCG) is the dominant form [13, 14]. It has been proposed that the presence of galloyl moieties in ECG and EGCG act as antimicrobials through direct binding with bacterial peptidoglycan layer and interferes with its biosynthesis [15].

Tea is grown in different countries and climates across the world under a rain-fed mono-cropping system that is influenced by climatic variations that determine optimal growth. Given the increasing drought conditions together with salinity there is a need for thorough investigation of molecular and physiological processes involved in salt and drought tolerance in order to improve the agronomic traits in tea [16, 17]. In recent years DNA delivery through *Agrobacterium*-mediated genetic transformation has been widely used for the production of transgenic plants to improve the agricultural and nutritional traits.

However, biotechnological exploitation and application of plant genetic resources for trait improvement and stress resistance enhancement in tea have been hindered due to the recalcitrance of tea plants to *Agrobacterium*-mediated genetic transformation, even though some genetic transformation of tea plants has been reported for some clones of *Camellia sinensis* var. *assamica* [18–20]. A well-established protocol for tea genetic transformation is required to overcome this bottleneck. During in vitro *Agrobacterium*-mediated tea transformation, the polyphenols released from the explant wounding site suppress the growth of *Agrobacterium* due to their bactericidal effect [21]. Generally, the high amount of bactericidal polyphenols is also toxic to the plant tissues, probably because of quinone formation from the oxidation of tannins and polyphenols following wounding or stress [22]. Although the polyphenols are less in calli compared to tea leaves [22, 23], the accumulation of phenolic content moderately increases during sub-culturing [24] and stress conditions such as explant excision, wounding, co-cultivation of explants with *Agrobacterium*, antibiotic selection, light exposure, and application of disinfectants for explant surface sterilization [22, 25, 26]. During in-vitro *Agrobacterium*-mediated transformation, a co-culture period of 2–3 days is usually required for most crop species. Whereas in tea an optimum period of 5–6 days is recommended to enhance the transformation efficiency [18, 19], which may cause excessive explant browning [26]. For optimizing the *Agrobacterium*–mediated tea transformation efficiency and to control phenolic oxidation of tea explant tissues, different culture conditions and media supplemented with different adsorbents and antioxidants have been tested to mitigate tissue browning (necrosis) and improve genetic transformation efficiency [26]. The oncogenes of root-inducing (Ri) plasmid from the extrachromosomal replicon of *A. rhizogenes* can result in the formation of independent hairy roots after being integrated into the plant genome [27]. Genetically transformed hairy roots can be generated from explants such as leaves and stems by *A. rhizogenes* infection for plant metabolic engineering, plant-pathogen interaction, nodulation, mycorrhization, and phytoremediation studies [28–32]. Transgenic hairy roots can be also induced from wild-type shoots, resulting in the production of composite plants [32]. This composite system allows for “in root” examination of transgene functions in the context of a complete plant and can be utilized for root-shoot interaction studies and crop trait improvement without changing its shoot genetic background. Thus, the offspring generated either by sexual or asexual means from wild type tissues of the composite plant should not contain any transgene.

To extend the study of genetic analysis and gene function, efficient transformation techniques are needed. The various explants of tea such as shoots, hypocotyl, cotyledon and cotyledonary nodes have been used to produce genetically modified plants either by using particle bombardment or *Agrobacterium*-mediated transformation. However, on larger scales these transformation methods are insufficient and labor-intensive. Genes involved in root biology are under investigation for nutrient uptake, pathogen interactions, symbiosis, hormone transport. These problems can be addressed using *A. rhizogenes*-mediated transgenic hairy root induction [33], which can be easily analyzed.

## Methods

### Plant materials and growth

Mature seeds were collected during autumn from 7-year-old tea plants (*C. sinensis var. sinensis* cv. “Nong Kangzao”) grown at the experimental tea farm of Anhui Agricultural University, Hefei, China. Seeds were soaked for overnight after rinsing with tap-water and removal of the outer coat. Using a strainer, the “floaters” and “sinkers” were separated. “Sinkers” were used as primary batch for sowing. The selected seeds were soaked in 4% bavistin (Guoguang Agricultural Chemicals, Si-Chuan, China) overnight to eliminate fungal contamination, followed by 70% (v/v) ethanol for 3 min and 0.1% mercuric chloride for 4 min and thoroughly rinsed five times in sterile distilled water. Seed sterilization could minimize the risk of microbial contamination and infection. Sterilization is essential when growing plants in a humid chamber since the warm, humid environment in the chamber promotes the growth of pathogens, especially fungi. The mixture of Pindstrup high quality peat substrate (Pindstrup Horticulture Ltd, Shanghai, China) and vermiculite (3:1 v/v) was placed in polythene bags and autoclaved for 15 min at 121 °C, 15 psi. The seeds were placed into wet autoclaved mixture in pots (12 cm in dimeter and 14 cm in height) for germination in a growth chamber at 26 ± 2 °C for 2 months.

### *Agrobacterium rhizogenes* strains

Wild-type *A. rhizogenes* A4 agropine-type strain obtained from Agricultural Culture Collection of China (ACCC) and its transformant containing the binary plasmid pBI121 (14.7 Kb) were used in this study. pBI121 contains *uidA* reporter gene (GUS, *β*-glucuronidase) driven by the cauliflower mosaic virus 35S promoter and the terminator of nopaline synthase (*nos*) gene. The binary vector was introduced into *A. rhizogenes* strains by electroporation [34] and transformants were selected on A4 solid media (10 g sucrose, 0.2 g MgSO_4_.7H_2_O, 0.5 g K_2_HPO_4_, 0.2 g CaSO_4_, 0.1 g NaCl, 1.0 ml of 1% NaMoO_4_, 1 ml of 1% C_6_H_5_FeO_7_, 1 ml of 1% Boric acid, 1.0 g Yeast extract and 15 g Agar in 1000 ml of double distilled H_2_O, pH 6.8–7.0) (Sinopharm Chemical Reagent Co. Ltd, Shanghai, China) supplemented with 50 mg/l kanamycin.

### Plant transformation

A single colony of A4-wild type and the A4 transformant harboring pBI121 was used to inoculate 5 ml of A4 liquid media. The cultures were incubated overnight at 28 °C with shaking (200 rpm) on an orbital shaker (Zhicheng, Shanghai, China). To develop enough bacterial paste both the strains were streaked on respective media and incubated for 4 days at 28 °C. It is critical to always inoculate *Agrobacterium* cultures on A4 sloid media directly from glycerol stocks and not from stored plates. For hairy root induction, 2 months old healthy seedlings were selected. The hypocotyl region was punctured using a needle carrying a drop of *Agrobacterium* paste (OD_600_ = 0.1) and the punctured region was smeared with *Agrobacterium* paste using bent glass rod. It is critical to smear the *Agrobacterium* paste with a bent glass rod around the wounded hypocotyl region to enhance infection. The infected plants were transferred to a humid chamber and watered with 10% A4 strain suspension with a culture density of OD_600_ = 0.6 from the first 2 weeks. After 2-weeks the plants were transferred to growth chamber and kept under 16 h light (cool white fluorescent light tubes providing irradiance of 40–50 mmol m ^−2^ s^−1^) and 8 h dark cycle at 26 ± 2 °C. The plants were periodically watered with 1% MS (Murashige and Skoog) medium and water for 3 months. Critical step: Watering of the seedlings with A4 suspension for the first 2 weeks is a key step to increase the *Agrobacterium* population in the pot mixture to enhance infection.

### Transgene analysis

The roots from infected and non-infected seedlings were collected and washed with running tap water for 1 h and rinsed with sterile distilled water. The roots were surface sterilized with 70% ethanol for 1 min to remove *Agrobacterium* and other microbes from the surface of roots. Genomic DNA was extracted from the sterilized roots using the MiniBEST Plant Genomic DNA Extraction Kit (TaKaRa, Dalian, China). PCR detection of transgenes in root DNA extracts were performed using gene specific primer pairs for *rol C* and *aux1* which are present in the T-DNA region of *A. rhizogenes*. Further PCR detection for *uidA* was carried out to confirm the transgene integration in roots transformed with A4 harboring pBI121, using gene specific primers (Table 1) as per manufacturer’s instructions (TransGene, Beijing, China). Programmable thermal cycler (Bio-Rad S1000) was used for the amplification under the following cycles: initial denaturation at 95 °C for 3 min, 30 cycles of amplification (95 °C for 30 s, 58 °C for 30 s and 72 °C for 1 min, and 72 °C for 10 min). PCR product was resolved on 1.5% agarose gel and stained with ethidium bromide for visualization of the bands. To check the expression levels of some root-inducing genes in the shoots of the composite plants, leaves were collected to extract total RNA using RNAprep pure Plant Kit (TianGen Biotech., Ltd, Beijing, China). The quality and quantity of RNA were analyzed using a Nanodrop 2000 spectrophotometer (Thermo Fisher Scientific, Wilmington, DE, USA) and agarose gel. Quantitative real-time PCR (qPCR) expression analysis was performed on a CFX96 platform (Bio-Rad, California, USA) using gene specific primers for *uidA* and six other *Agrobacterium* genes *rol A*, *rol B*, *rol C*, *rol D*, *ORF13a*, and *ORF14* with 18S rRNA as reference gene for data normalization (Table 1).

**Table 1:** Primers used in this study

Primer code	Sequence of the primer	Note
*rol a*	5′-atggaactagccggaataaa-3′ 5′-accccgtaggtctgaatatt-3′	qPCR analysis
*rol b*	5′-atggcactgaacttgccgtt-3′ 5′-agtcgccgaggtttctttct-3′	qPCR analysis
*rol c*	5′-atggcggaatttgacctatg-3′ 5′-ctccattccaaatttgcatt-3′	qPCR analysis
*rol d*	5′-atggctcgttatttcggcag-3′ 5′-ttccaacaggaccttgccaa-3′	qPCR analysis
*orf13a*	5′-atgctcaccgctcacgcgat-3′ 5′-ggtgttccaaattgactggc-3′	qPCR analysis
*orf14*	5′-atgagcatggcagatgagtt-3′ 5′-aaatacactctttccagcac-3′	qPCR analysis
*uidA*	5′-attggggccaactcctaccgtac-3′ 5′-gctgctgtcggctttaacctctct-3′	qPCR analysis and gene detection
*rol c*	5′-tgtgacaagcagcgatgagc-3′ 5′-gattgcaaacttgcactcgc-3′	PCR gene detection
*aux1*	5′-ccaagcttgtcagaaaacttcaggg-3′ 5′-ccggatccaatacccagcgcttt-3′	PCR gene detection

### GUS assay

The histochemical assay for the reporter gene GUS activity was performed using the established method [35]. For histochemical detection, the transformed roots were incubated overnight in a solution containing 25 mg/l of histochemical substrate 5-bromo-4-chloro-3-indolyl glucuronide (Aladdin, Shanghai, China), 10 mM EDTA, phosphate buffer, 0.1% Triton X-100 and 20% methanol, pH 8.0. The reaction mixture was placed under mild vacuum for 5 min and incubated overnight at 37º C. After incubation, blue color was observed on root tissues transformed with A4 strain carrying pBI121. No color was observed on A4-wild type strain transformed hairy roots.

## Results

### High efficiency protocol for the generation of composite plants in tea

**Fig. 1:**
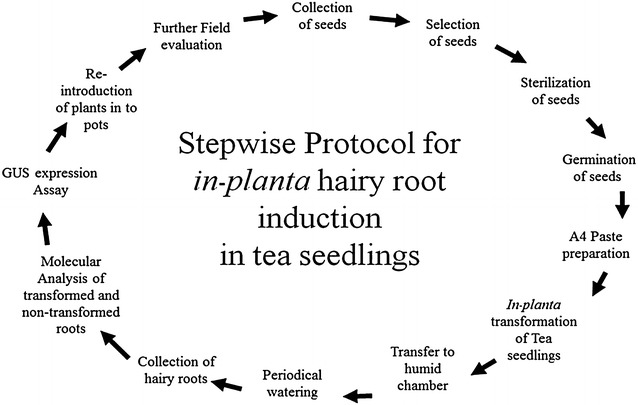
The stepwise protocol for *in*-*planta* hairy root induction in tea seedlings

The use of *ex vitro* composite plants through *A. rhizogenes*—mediated transformation has been widely researched in various plant species [36–38]. We have optimized the hairy root induction protocol in tea (Fig. 1; Table 2) that reliably generates high efficiency transformed roots suitable for root biology studies. The surface sterilized seeds were transferred in polypropylene pots containing high quality Pindstrup substrate and allowed for germination at 26 ± 2 °C for 2-months. The healthy seedlings were selected for hairy root induction to produce composite plants. The 2-month old seedlings were carefully up-rooted from the substrate and washed with running tap water for 10–15 min. The seedlings were infected as described in methodology and transferred in mini plastic acrodomes and watered with 10% A4 strain suspension with a culture density of OD_600_ = 0.6 for 2 weeks. Among the factors that enhance high efficiency hairy root induction, we found that smearing of *Agrobacterium* paste around the punctured region, maintaining humid conditions after infection and continuous watering with A4 suspension culture at a density of OD_600_ = 0.6 were essential. We also observed root initiation at the punctured region of hypocotyl after 2-months of infection. Compared to control plants without A4 infection (Fig. 3a–c), 88.3% (± 2%) of infected plants with A4-wild type and A4-harboring pBI121 produced transgenic hairy roots (Table 3; Fig. 3d–g). After 5 months the roots were collected from the non-infected, A4-wild type and A4-harboring pBI121 infected plants to confirm the integration of T-DNA region in the transformed hairy roots. The presence of *Agrobacterial* transgenes *rolC*, *aux1* and *uidA* were PCR detected in the hairy roots, thus confirming successful gene transformation (Fig. 4a, b). To further validate the presence of the marker enzyme GUS, transformed hairy roots of A4-wild type (control) and A4-harboring pBI121 were used for histochemical GUS staining. Development of deep blue color in transgenic hairy roots, following histochemical staining for GUS activity further confirmed the transgenic status of hairy roots tested (Fig. 5). Furthermore, qPCR analysis revealed that none of the transgenes were expressed in the leaves of the composite plants (Fig. 4c). For *rolD* and *uidA*, their expression ratios to the reference gene 18S rRNA were only 0.00029 and 0.00081, which were likely resulted from non-specific amplification.

**Table 2:** Troubleshooting

Problem	Reason	Solution
Poor germination and seedling growth	Used floaters Poor storage of seeds	Use sinkers Use desiccants during storage at 4 °C
Lack of induction of hairy roots	*Agrobacterium* may lose virulence over the culturing process or become contaminated Seedling viability is too low Plants were not periodically watered	Use fresh − 80 °C glycerol stock Grow new seedlings Water plants regularly

**Table 3:** Effect of *Agrobacterium rhizogenes* (A4) on hairy root induction in tea seedlings

Number of seedlings	Number of seedlings with hairy root	PCR *rolC*	PCR *aux1*	Final transformation efficiency (%)
20	18	+	+	90
20	18	+	+	90
20	17	+	+	85

Average of final transformation efficiency (%) 88.33

‘+’ indicates the presence of *rolC* and *aux1* in transformed roots

## Discussion

The in-vitro transformation of tea faces many challenges limiting the realization of a viable and feasible protocol for tea transformation. *In*-*planta Agrobacterium* transformation gives a practical alternative in overcoming challenges that arise from in-vitro transformation such as meticulous sterilization, laborious plant management, and low transformation efficiency. This technique can be used for “in root” gene functional studies such as understanding of root-produced l-theanine metabolic pathways, shoot–root interaction, root resistance against different biotic and abiotic stresses. The improvement of the whole composite plant system resulting from the root genetic manipulation will not change the genotype of the composite plant shoots genetically, so that the economically important genetic traits of the shoots may be maintained, even enhanced.

In the present study, for *in*-*planta* hairy root induction in recalcitrant tea plants, the *A. rhizogene* strains A4 and A4-harboring pBI121 were found to be effective to establish chimeric plants with transgenic hairy roots at the hypocotyl proximal region. Our data indicated that using this protocol high-efficiency of hairy root formation (88.3%) in tea has been achieved. Further efforts should be made to improve this protocol such as *Agrobacterial* infection to obtain more consistent hairy root production among infected seedlings. Similar *in*-*planta* transformation has been reportedly applied to generate transgenic hairy roots in recalcitrant bean species, *Phaseolus acutifolius, P. vulgaris, P. coccineous, P. lunatus* for various purposes [39]. Similarly, Vieweg et al. [40] demonstrated the effective induction of hairy roots for DNA transfer using an agropine type *A. rhizogenes* strain on the model legume *Vicia hirsuta, V. faba, Medicago truncatula* and *Pisum sativum*. These previous findings suggest that the hairy roots can be induced on a wide range of legumes with appropriate *A. rhizogenes* by infecting the hypocotyl and/or the cotyledonary node. Here, in our current protocol an attempt has been made to transform tea plant, with an emphasis to reduce oxidative browning. This protocol may serve as an efficient tool for the rapid validation of transgene and tea root biology studies. Based on the efficiency of this protocol we might possibly achieve similar transformation efficiency with many other tea varieties for practical and biological study purposes.

## Conclusion

The protocol described here has been successfully adapted to induce hairy roots in the recalcitrant Chinese tea variety “Nong Kangzao”. *In*-*vitro* transformation has more challenges, which limits success in improving tea varieties; *in*-*planta* transformation gives an alternative in overcoming challenges posed by in-vitro transformation. This protocol is an important tool for the study of root biology and secondary metabolites. It may be possible to achieve similar efficiency with all tea varieties, other woody and medicinal plants.
